# Diseased Germs

**Published:** 1876-08

**Authors:** 


### EDITORIAL, ETC.

*Diseased Germs.*—The *Sanitarian* thus describes an affection of the mouth, to which but slight attention is generally given:

Mothers know too well what is meant by the word "thrush," or "sprew," that mouth malady too common with little children. To the profession it is known as an aphthous ulceration of the tongue, aphtha being the name of the disease, and signifying a burning. The tongue "is swollen, tender and furred." There are excoriated spots, sometimes true ulcers, varying in size, perhaps, from that of a pin's head to that of a half pea, and these are severally capped with a white curd-like mass. However diminutive these pustules may be, they are in truth hammocks of tiny plants, for each one contains many thousands of parasitic fungi often called *torula.* These fungi attach themselves to the mucous membrane, and burrow among the epithelial cells. They are "composed of threads matted together like felt," whose basal ends interwine among the epithelia, like hair in the prepared mortar of the plasterer. At a recent meeting of the Academy of Natural Sciences, Professor Leidy exhibited a mouse with little curdy patches on its ears, face and nose. Mr. Indifference would have passed the matter by as a stupid trifle; and a spurt of insapience escaped one of the wise men, who wished to know "what the muss was." However, little *Mus musculus* was regarded as an abnormal case, and a proper subject of scientific inquiry. The query was now, "What ailed the little fellow, and where had he been?" At this juncture the microscope spoke out in meeting, declaring with authority that the white spots were colonies of a parasitic fungus; and, strange to tell, they were as much like the thrush fungus as one pea is like its fellow in the same pod. The truth told, Mousie was captured in the children's department of Blockley Hospital, where he had picked up the crumbs that had fallen from the mouth of a child patient. The diagnosis now seemed natural and direct. Mousie had been and got it—namely, the thrush—and strange to say, he had got it bad, for it was on his ears and nose and face. Soon, in all probability, it would have entered the mouth, even if it had not already. A minute portion of one of these white spots was subjected by skilled hands to a lens of very high power, and lo! there were the morbid parasites, their sporular bodies, some single, some double, and others "in chains of a dozen or more." The fungus was pronounced to be a *torula* or *oidium*, like that found in the disease known as thrush or aphtha. A drawing of it would simply be like a number of elongated beads strung together. But how diminutive these beads or cells were! A single one was the 1-650 of a line in length, that is, it would take 7,800 of them in line to make an inch.

